# Autoimmune encephalomyelitis in NOD mice is not initially a progressive multiple sclerosis model

**DOI:** 10.1002/acn3.792

**Published:** 2019-07-15

**Authors:** David Baker, Erik Nutma, Helen O'Shea, Anne Cooke, Jacqueline M. Orian, Sandra Amor

**Affiliations:** ^1^ BartsMS Blizard Institute Barts and the London School of Medicine and Dentistry Queen Mary University of London London E1 2AT United Kingdom; ^2^ Department of Pathology Amsterdam UMC, Location VUmc Amsterdam 1081HV The Netherlands; ^3^ Department of Pathology University of Cambridge Cambridge CB2 1QP United Kingdom; ^4^ La Trobe Institute of Molecular Sciences La Trobe University Bundoora Victoria 3086 Australia; ^5^ Present address: Department of Biological Sciences Cork Institute of Technology Cork Ireland

## Abstract

**Objective:**

Despite progress in treating relapsing multiple sclerosis (MS), effective inhibition of nonrelapsing progressive MS is an urgent, unmet, clinical need. Animal models of MS, such as experimental autoimmune encephalomyelitis (EAE), provide valuable tools to examine the mechanisms contributing to disease and may be important for developing rational therapeutic approaches for treatment of progressive MS. It has been suggested that myelin oligodendrocyte glycoprotein (MOG) peptide residues 35‐55 (MOG ^35‐55^)‐induced EAE in nonobese diabetic (NOD) mice resembles secondary progressive MS. The objective was to determine whether the published data merits such claims.

**Methods:**

Induction and monitoring of EAE in NOD mice and literature review.

**Results:**

It is evident that the NOD mouse model lacks validity as a progressive MS model as the individual course seems to be an asynchronous, relapsing‐remitting neurodegenerative disease, characterized by increasingly poor recovery from relapse. The seemingly progressive course seen in group means of clinical score is an artifact of data handling and interpretation**.**

**Interpretation:**

Although MOG ^35‐55^‐induced EAE in NOD mice may provide some clues about approaches to block neurodegeneration associated with the inflammatory penumbra as lesions form, it should not be used to justify trials in people with nonactive, progressive MS. This adds further support to the view that drug studies in animals should universally adopt transparent raw data deposition as part of the publication process, such that claims can adequately be interrogated. This transparency is important if animal‐based science is to remain a credible part of translational research in MS.

## Introduction

Multiple sclerosis (MS) is an immune‐mediated, demyelinating disease of the central nervous system (CNS).^1^ This typically follows a relapsing‐remitting disease course often followed by the accumulation of progressively worsening neurological disability.^1^ Active neurological disease in MS 2 and disease in the experimental autoimmune encephalomyelitis (EAE) model of MS is driven by the consequences of the peripheral, adaptive immune response entering the CNS.^3^ This is supported not only by radiological and pathological findings, but most importantly, by the response to therapy.^4^, ^5^, ^6^, ^7^ Although progressive MS may also respond to similar immunotherapy provided there is sufficient neurological reserve in the nerve‐tracks affected,^8^, ^9^, ^10^ other factors such as innate immune responses are thought to be of central importance in progressive neurodegeneration.^11^ This concept underpins the perceived treatment‐failure of immunotherapy in advanced (progressive) MS, where replacement of peripheral immunity, which stops relapses, does not always halt accumulation of disability.^8^, ^9^, ^12^, ^13^ Therefore, there is an urgent need for model systems that can be used to identify the pathological mechanism operating in progressive disease as well as to design and test new therapeutics.

Experimental autoimmune encephalomyelitis in animals is a group of experimentally induced autoimmune diseases with some similarities to MS.^14^, ^15^ Some of the EAE models are associated with the development of relapsing, immune‐mediated demyelination disease^14^, ^16^ and may show slow accumulation of disability that is independent of relapses and peripheral autoimmunity.^17^, ^18^, ^19^, ^20^ The nonobese diabetic (NOD) mouse develops spontaneous or induced diabetes and other endocrine gland‐associated autoimmunities.^21^, ^22^, ^23^ This mouse strain has also been reported to develop progressive neurological disease that mimics progressive MS.^24^, ^25^ Progressive worsening appears to develop within 20–30 days postinduction following immunization with myelin oligodendrocyte glycoprotein (MOG) peptide residues 35‐55 (MOG^35‐55^).^24^, ^25^, ^26^, ^27^, ^28^, ^29^, ^30^, ^31^ Thus, this EAE model could have significant utility for screening potential neuroprotective and repair agents.^24^, ^27^, ^28^, ^29^, ^30^, ^31^ Here, we have investigated the response of NOD mice to various autoantigens to assess the validity of this system to model progressive MS. In contrast to some of the published literature indicating the progressive nature of early MOG^35‐55^ induced EAE in NOD mice, we found no evidence that this model exhibits a progressive worsening independent of relapsing disease indicating that existing data are an artifact of data handling and interpretation. Therefore, this model should not be used to justify any human trials in nonrelapsing progressive MS.

## Methods

### Animals

NOD/Lt (NOD), NOD.H2Ea (NOD‐E) mice expressing a transgenic H‐2A alpha chain allowing expression of H‐2E^g7^ and NOD.H2Ab^Asp^ (NOD‐ASP) mice expressing a modified H‐2A beta with a serine to aspartic acid substitution at position 57 were from stock bred at the University of Cambridge.^22^ These mice failed to develop diabetes during the course of these studies. Animals were housed and used according to the Animals (Scientific Procedures) Act 1986, which induces review by the local Animal Welfare and Ethical Review Body and the United Kingdom Government, Home Office Inspectorate. In addition, NOD/ShiLtJ mice were from stock bred at the La Trobe University.^32^ Animal procedures were approved by the Institutional Animal Care and Use Committee at the La Trobe Institute, Australia.^23^, ^32^

### Experimental autoimmune encephalomyelitis

Animals (11–15 week) were injected subcutaneously with 1 mg mouse spinal cord homogenate (SCH),^16^ 200 *μ*g mouse proteolipid protein residues 56‐70 (PLP^56‐70^) peptide^33^, ^34^ or 200 *μ*g MOG residues 8‐22 (MOG^8‐22^) peptide^34^, ^35^ emulsified in Freunds adjuvant supplemented with 60 *μ*g *Mycobacteria tuberculosis* and *M.butyricum* on day 0 and 7.^16^ Animals were injected intraperitoneally with 200 ng *Bordetella pertussis* toxin immediately and 24 h after each injection of antigen, as described previously.^16^, ^32^, ^33^ These are the immunodominant myelin peptides associated with H‐2A^g7^ reactivity in ABH mice.^33^, ^34^, ^35^ Animals were scored as 0 = normal; 1 = limp tail; 2 = impaired righting reflex; 3 = hindlimb paresis; 4 = hindlimb paralysis; 5 = moribund (endpoint) as described previously.^16^, ^33^ Alternatively, animals (9–12 week) were immunized with 200 *μ*g MOG residues 35‐55 (MOG^35‐55^) peptide emulsified in Freunds complete adjuvant supplemented with 4 mg/mL *M. tuberculosis* on day 0 and 350 ng intravenous *B. pertussis* toxin on day 0 and day 2.^23^, ^32^ These mice were scored as: 0 = no signs, 1 = limp tail, 2 = hindlimb weakness, 3 = hindlimb weakness with at least one paralyzed hindlimb, 4 = paralysis of both hindlimbs and weakness of one forelimb, 5 = moribund.^23^ Animals were randomly assigned to treatments and the studies were scored blinded to induction agent. Groups contained a minimum of five animals/group which was sufficient to perform statistical analysis and experimental elements relevant to the ARRIVE guidelines have been reported previously.^13^, ^15^ Raw data supplied as Data S1.

### Statistical analysis

Data were analyzed using Sigmplot (Systat Software Inc, London) and expressed as mean ± standard error of the mean. For EAE group clinical scores and day of disease onset were assessed by Mann–Whitney U tests. Group EAE score represents the maximal neurological deficit in all animals within the group and mean EAE score the maximal neurological deficit developed by mice that exhibited EAE, as previously described.^33^, ^36^ *P* values < 0.05 were considered significant.

## Results

**Figure 1:**
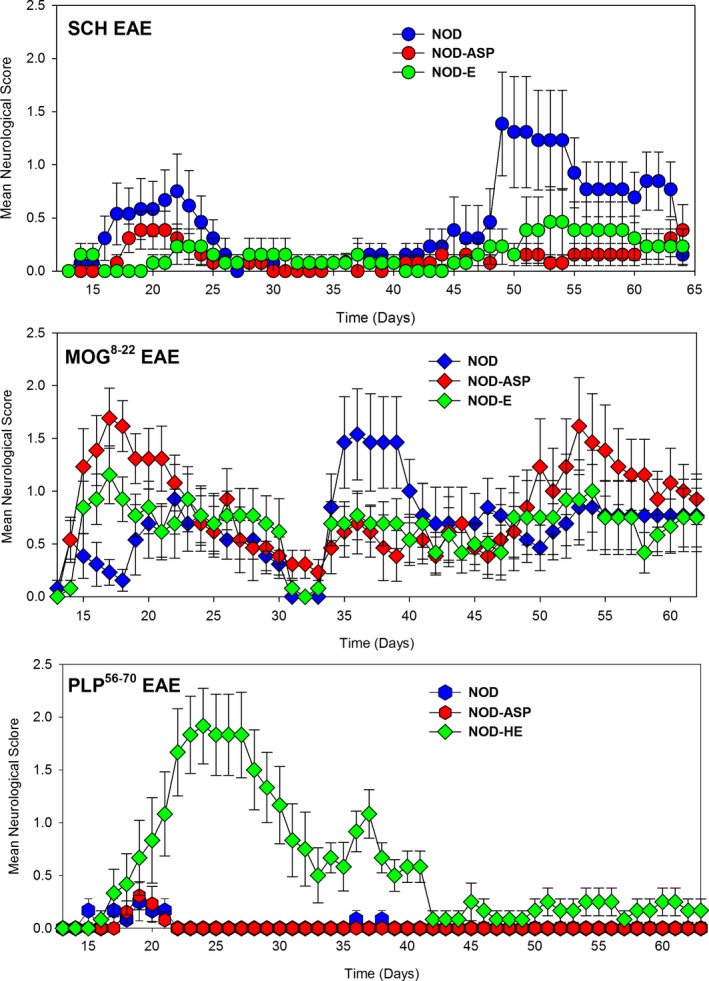
Induction of EAE in NOD mice using myelin peptides. EAE was induced by subcutaneous injection of neuroantigen in Freunds adjuvant and using *B.pertusssis* toxin as coadjuvant. The results represent the group mean daily ± SEM.

Biozzi ABH mice (H2^dq1^: K^d^, A^g7^, E^‐^, D^q^) are susceptible to a number of induced autoimmunities^37^ exhibit high‐susceptibility to SCH‐induced EAE compared to NOD (H2 ^g7^:K^d^, A^g7^,E^‐^, D^b^) mice, which share the diabetogenic H‐2A^g7^ molecule.^37^, ^38^ However, NOD mice can show comparable susceptibility to other induced autoimmunities when *B. pertussis* toxin is used as coadjuvant.^39^ The immunodominant epitopes associated with the development of EAE in ABH mice are proteolipid protein (PLP^56‐70^) and myelin oligodendrocyte glycoprotein (MOG^8‐22^) peptides.^33^, ^34^ However, wild‐type NOD mice only exhibited modest susceptibility (*n* = 9/13) to SCH‐induced EAE (Fig 1; Table**** 1), with disease that had poor consistency in severity (range of maximum severity grade 0.5–4 *n* = 9) and day of onset (range 14–49. *n* = 9). Of those mice that developed disease 6/9 developed relapses by day 63 and none showed a slow progressive worsening of disease. Similarly, MOG^8‐22^ peptide induced disease in 13/14 mice, which largely relapsed (*n* = 9/13), and again no escalating progressive disease was evident. Interestingly, it was found that wild‐type mice poorly responded to PLP^56‐70^ peptide and only 5/13 (38.5%) mice developed low‐grade EAE (Table 1). Likewise, NOD.ASP mice developed low‐grade EAE (Fig. 1). However, NOD‐E mice exhibited significantly (*P* < 0.001) more severe disease than wild‐type mice immunized with PLP^56‐70^, as described previously.^40^ This was evident when the group score (2.1 ± 0.3 vs. 0.5 ± 0.2; *P* < 0.001) and the EAE score (2.3 ± 0.3 vs. 1.2 ± 0.2; *P* < 0.05) were analyzed (Table 1). Although some NOD‐E mice relapsed, there was no evidence of progressive worsening.

**Table 1:** Disease susceptibility of transgenic nonobese diabetic mice to myelin antigens

Strain	Immunogen	No. EAE	Group Score	EAE Score	Day of Onset
NOD	SCH	9/13	2.0 ± 0.5	2.8 ± 0.5	27.1 ± 14.9
NOD	MOG^8‐22^	13/14	1.5 ± 0.3	1.7 ± 0.3	17.3 ± 6.4
NOD	PLP^56‐70^	5/13	0.5 ± 0.2	1.2 ± 0.2	20.6 ± 9.3
NOD‐E	PLP^56‐70^	11/12**	2.1 ± 0.3***	2.3 ± 0.3*	21.6 ± 4.7
NOD	MOG^35‐55^	14/14	2.3 ± 0.1^#^	2.3 ± 0.1^#^	13.2 ± 3.1

EAE was induced by subcutaneous injection of neuroantigen in Freunds adjuvant and using *B. pertusssis* toxin as coadjuvant. The results represent the mean maximum group score of the first episode ± SEM; the mean maximum score of animals that developed EAE during the first episode ± SEM and the mean day of onset ± SD. The NOD mice immunized with SCH, MOG^8‐22^ or PLP^56‐70^ were from stock based in the United Kingdom. The NOD mice immunized with MOG^35‐55^ peptide were from different stock based in Australia and #the scoring system used was different.

*P < 0.05; **P < 0.01, ***P < 0.001 compared to wild‐type mice.

Previous studies have examined the encephalitogenic response to MOG^35‐55^ in NOD mice.^24^, ^41^ This is a subdominant MOG peptide in both the ABH (H‐2 ^g7^) and C57BL/6 (H‐2^b^) mice.^34^, ^42^ This peptide tends to induce a monophasic chronic EAE in ABH and C57BL/6 mice, where there is poor recovery following a single attack.^42^, ^43^, ^44^ This is consistent with the neurodegenerative nature of the attacks.^45^ Therefore, the effect of MOG^35‐55^ peptide was not assessed in the initial studies (Fig. 1). An apparent progressive worsening of neurological signs can be observed when analyzing the group means of ABH animals with SCH‐induced EAE,^16^ yet it is clear that in individual mice the course of disease is relapsing‐remitting that responds to T‐cell immunotherapy.^16^, ^17^, ^46^ The apparent continuing worsening of disease is simply due to the occurrence of asynchronous relapses with increasingly poor recovery due to the neurodegenerative effects of the inflammatory penumbra.^16^, ^19^, ^20^, ^46^ This may explain the disease course reported in NOD mice.^24^, ^25^, ^26^, ^27^, ^28^, ^29^, ^30^, ^31^

**Figure 2:**
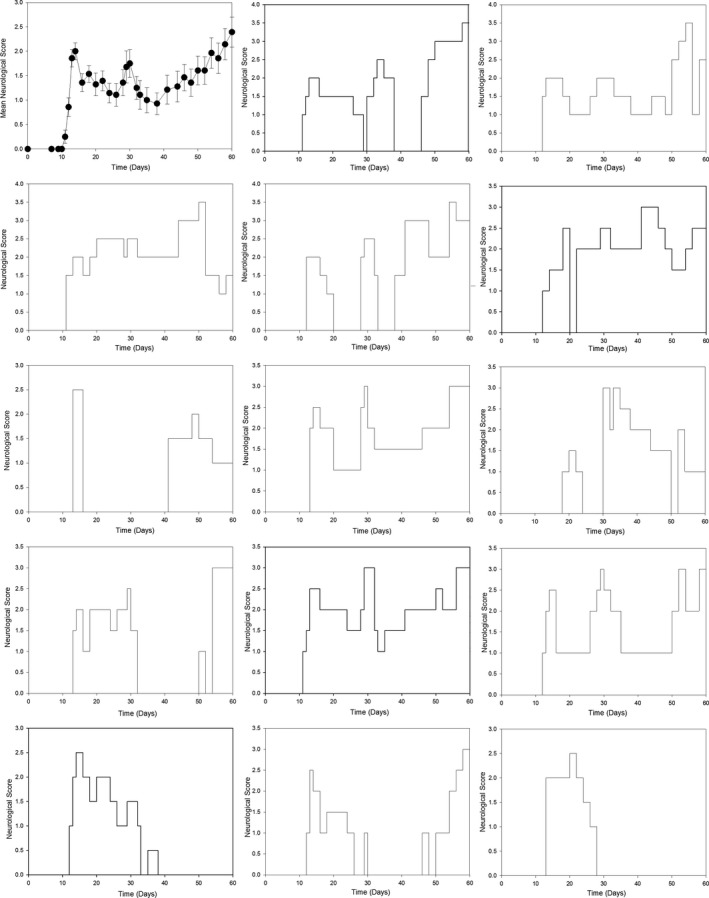
Individual disease courses in NOD mice. NOD mice were immunized with 200 *μ*g MOG ^35‐55^ and 4 mg/mL complete Freunds adjuvant. The results represent the group mean ± SEM neurological score (*n* = 14) and the individual scores over time.

**Figure 3:**
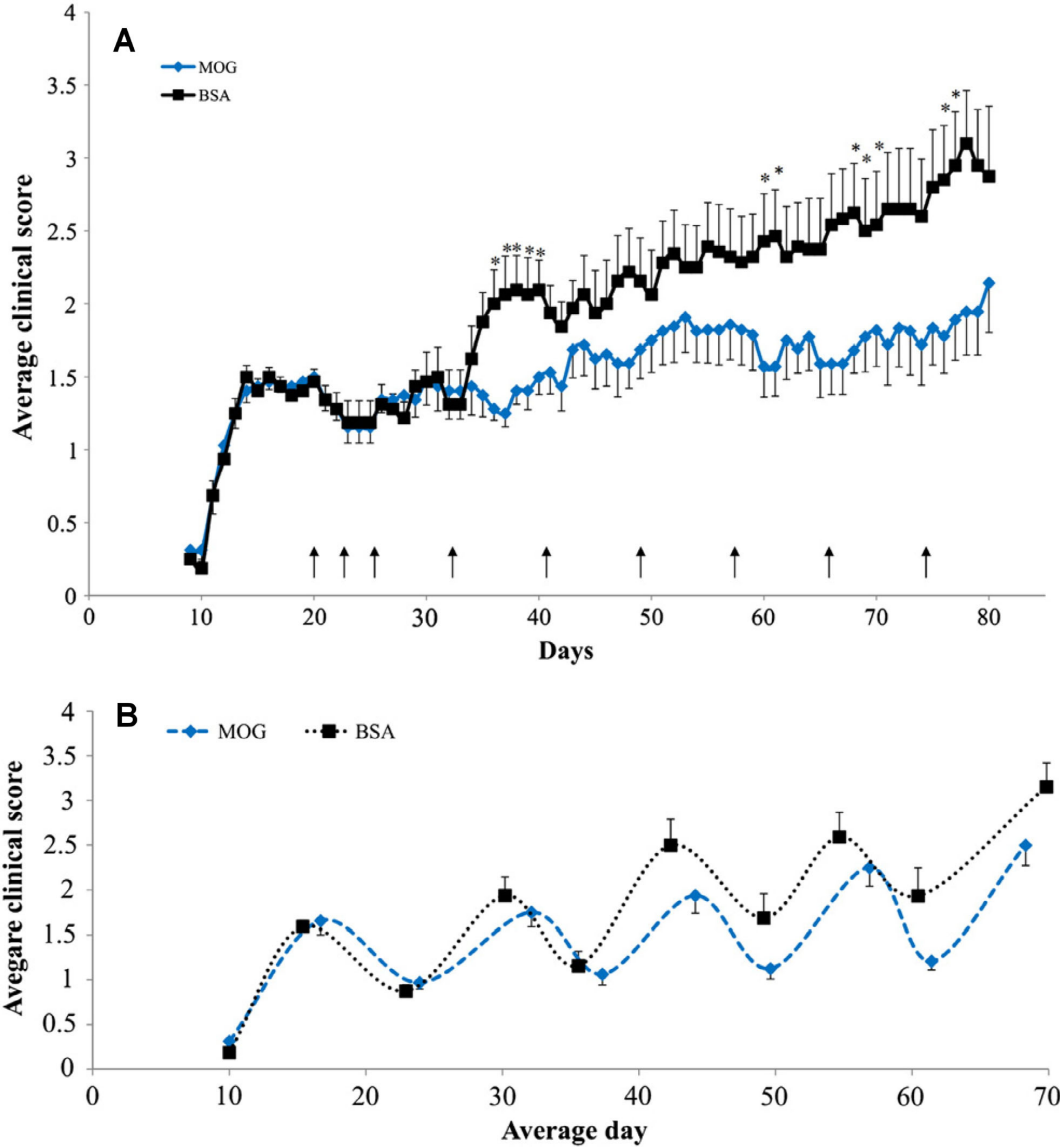
Progressive worsening of EAE disease in NOD mice is a misrepresentation of relapsing‐remitting EAE. NOD mice were immunized with 150 *μ*g MOG ^35‐55^ and 4 mg/mL complete Freunds adjuvant in the progressive EAE model. (A) The results demonstrating a progressive worsening was shown using a mean daily group score ± SEM. These mice were treated (arrow) with MOG ^35‐55^ nasal tolerance or bovine serum albumin (BSA) peptide as control. Differences between the groups are shown. *P < 0.05 assessed using a Student's t test. While a t test is not appropriate for such nonparametric data,^3^, ^68^ the lack of consistent statistical differences demonstrates the fluctuating nature of the individual data points that form the group score. (B) As each mouse had a relapse and remission at different time points, the average clinical score of each relapse and remission was calculated, clearly showing disease is relapsing remitting.^26^ Figures are reproduced from Levy Barazany H et al. Exp Neurol 2014; 255:63‐70^26^; doi.org/10.1016/j.expneurol.2014.02.010 with permission from Elsevier.

Therefore, to avoid unnecessary use of animals, the literature was investigated further. Indeed, the first descriptions of MOG^35‐55^ reported that the disease in NOD mice was relapsing and remitting.^23^, ^41^, ^47^ Importantly, when the data from individual animals are examined it is clear that disease was largely relapsing and remitting and not progressive^48^ and was confirmed here using available data (Fig. 2). Thus the apparent progressive worsening was largely due to asynchronous relapses with poor recovery (Fig. 2). The relapsing nature of EAE in NOD mice was reproducibly supported by the results from different laboratories.^23^, ^47^, ^48^, ^49^ Indeed, the literature (Fig. 3) indicates that defining MOG‐induced EAE in NOD mice as progressive, is a misinterpretation and misrepresentation of what actually is a neurodegenerative, relapsing disease profile.^26^, ^45^

## Discussion

This study demonstrates that MOG‐induced EAE in NOD mice induces a relapsing disease course that is not reflective of chronic, progressive MS in its early phases. This is consistent with the original description of MOG^35‐55^‐induced disease in NOD/Lt mice as being a relapsing‐remitting disease.^41^ However, when mice were followed for up to several months longer it was reported that disease might become chronically progressive as animals accumulate disability.^47^ However, with the urgent unmet clinical need to develop treatment options for advanced MS, MOG^35‐55^ induced EAE has become used as a “progressive EAE” model that purportedly resembles secondary progressive MS.^24^, ^25^, ^48^ This clinical progression appears to begin from about day 20 after immunization when it is used to test therapeutics for use in progressive MS.^24^, ^25^, ^26^, ^27^, ^28^, ^29^, ^30^, ^31^ However, through literature review and as shown here, the same clinical course has been reported to be a form of chronic relapsing EAE.^23^, ^48^, ^49^, ^50^ At the cellular level, CD4 T cells from MOG^35‐55^ T‐cell receptor (TCR^MOG^)‐specific transgenic NOD mice, that select CD4 and CD8 T cells, induce a phenotypic progressive disease in NOD.*Scid* mice, similar to that reported using MOG^35‐55^ immunized wild‐type NOD mice.^51^ However, based on the disease occurring following active immunization in NOD. TCR^MOG^ mice, it is clearly evident that a relapsing‐remitting course developed.^51^ Indeed, the initial disease was described to be of mild severity that completely resolved followed by relapse of greater severity that did not resolve completely.^51^ Histologically, every attack is associated with the development of severe immune‐infiltration^52^ and thus lacks evidence of progression without significant blood–brain barrier dysfunction similar to progressive MS.^53^ Importantly, the pathology demonstrates that these immune attacks cause significant neurodegeneration, consistent with EAE in other mouse strains including C57BL/6 mice,^16^, ^36^, ^45^ leading to persistent disability that often increases with each cycle of neurological attack. Thus the concept of progressive worsening in the clinical score of NOD mice, is a misrepresentation of what is clearly an asynchronous‐neurodegenerative, relapsing EAE.

Such relapses, driven by T cells^17^, ^51^ will of course respond to immunotherapy. This could limit the generation of neurodegeneration by prevention of lesion formation or by inhibiting the consequence of the inflammatory penumbra that is associated with the formation of lesions in the central nervous system.^20^, ^45^, ^54^ Indeed, MOG^35‐55^ induced NOD mouse EAE responds to prophylactic and therapeutic T‐cell tolerance induction and T‐cell immunotherapy.^26^, ^27^ However, that these approaches have largely failed to markedly influence nonactive, progressive MS, demonstrates that the model as used, probably has no or limited validity and predictive value for efficacy in nonactive, progressive MS.^12^, ^13^, ^55^, ^56^ Therefore, the model should not be used to justify any clinical trials in nonrelapsing, progressive MS.^2^

Any agent that diminishes the frequency of attacks and reduces their severity will be potentially secondarily neuroprotective, as seen in both EAE and MS.^3^, ^57^ In addition, the inflammatory penumbra occurring during active EAE and MS is damaging and can cause nerve loss.^45^, ^54^, ^58^, ^59^ As such, it is sometimes difficult to dissociate direct neuroprotective effects against the inflammatory penumbra from secondary neuroprotection due to immunomodulation that prevents lesion formation. This is particularly the case when treating during active paralytic disease and monitoring recovery,^3^, ^54^, ^60^ such as that occurring in monophasic, neurodegenerative MOG‐induced EAE in C57BL/6 mice.^43^, ^61^ Although one can show that agents are not immunosuppressive in vitro, drugs that interfere with nervous system signaling can cause adverse effects in vivo, although these are seldom reported in animal studies.^3^, ^60^, ^62^ Such drugs that interfere with neuronal signaling could induce a stress response that could be immunosuppressive. Thus, it is imperative that these influences are avoided if insight into neuroprotection is required.^3^, ^60^, ^62^ This is because, while immunomodulation of the adaptive immune response may be of benefit for active progressive MS^3^, ^8^, ^9^, ^10^ it cannot adequately inform about effects operating in nonactive progressive MS.^2^ Until this aspect is appreciated by experimental biologists and clinicians, we will fail to adequately model progressive MS and continue to fail to translate ideas into human benefit.

Although not reported here, our previous studies have reported the histological profile of EAE in NOD mice.^22^ This is consistent with other mouse strains that demonstrate a dynamic degree of adaptive immune cells infiltration as clinical disease develops and wanes.^22^, ^36^ Glial cell inflammation is thought to be part of the substrate for slow progressive nerve loss in MS.^11^ However, the loss of axons and myelin, accumulation of microglial activation and gliosis reported in progressive EAE models,^24^, ^25^, ^26^, ^27^, ^28^, ^29^, ^30^, ^31^ is not qualitatively different from that found following the accumulation of disability from relapsing EAE in mice.^20^, ^36^ This perhaps is not surprising as monophasic or relapsing inflammatory disease activity probably creates and conditions the neurodegenerative environment that leads to the slow loss nerve loss, which does not respond rapidly to agents that target relapsing disease.^17^, ^18^, ^19^, ^20^ Progressive neurodegenerative pathology, driven by glial cells, initially coexists with adaptive immune inflammation driving active attacks, but becomes more dominant with disease duration as relapses wain, as occurs in NOD mice with long disease duration.^22^, ^47^

Given the deficits that NOD mice accumulate due to relapsing attacks, it is likely that slow progressive disease eventually develops, as a similar disease course is observed in SCH‐induced EAE in ABH.^17^, ^18^, ^23^ Following accumulation of deficit from relapsing attacks, animals exhibit slow clinical worsening and nerve loss, weeks to months after disease induction, which is not responsive to peripheral immunosuppression.^17^, ^18^, ^19^, ^20^ Although this degenerative process is occurring following initial attacks,^20^ this becomes notably evident during the postattack period that occurs following monophasic EAE that occurs in MOG^35‐55^‐induced EAE in C57BL/6,^43^, ^44^ chronic relapsing EAE in ABH^17^, ^18^ and possibly NOD mice.^23^, ^47^ It is possible that subtle differences in the genetics of the animals, age, sex, microbiome content, and breeding facility could account for the differences in the clinical profile reported here and as shown previously by us^23^ and that reported by others.^24^, ^25^, ^26^, ^27^, ^28^, ^29^, ^30^, ^31^ Indeed, in some of our studies, male and older mice tolerate inflammatory insults less well and accumulate nerve loss and deficits leading to the slow accumulation of progressive disability, even following a single attack.^54^, ^63^, ^64^ In our experience this deficit accumulates slowly^17^, ^18^ and was reported to occur in NOD mice months, not weeks after disease development.^23^, ^47^ Age and sex, however, are unlikely to account for differences in the clinical course observed here, as they were comparable^23^ to those reported for rapidly evolving secondary progressive EAE.^23^, ^24^, ^25^, ^26^ In our experience, this progressive worsening is not readily captured by the typical subjective, nonlinear, scoring of paralysis of the hindlimbs and tail often used to assess the severity of EAE in animals.^16^, ^23^, ^43^, ^47^ As such, neurological disease eventually plateaus in mouse EAE and remains stable over months.^18^, ^23^, ^43^, ^46^, ^47^ This may change very slowly just as observed in humans.^8^, ^17^, ^65^ However, other objective outputs such as spasticity and mobility changes can detect slow, worsening over time.^17^, ^18^ Similarly, although cuprizone‐induced demyelination and subsequent neurodegeneration was widely assumed to be nonclinical, through analysis of alternative objective outcome measures clinical deterioration can be detected.^64^, ^66^ Therefore, it may be possible that existing models, if used wisely, such as avoiding treatment during the periods of active attacks, could be used to identify candidate agents that may be of value in controlling nonrelapsing progressive MS. Alternatively models that mirror aspects of progressive MS can be developed to facilitate a mechanism‐based targeting of neurodegenerative disease in MS.

The process of the refinement, reduction, and replacement of animal use in research, which defines the ethical use of animals in research, means that animal experiments should have value in uncovering human biology and we should particularly strive to limit the number of animals used in severe procedures. EAE is such a severe procedure and thus should not be used if less‐severe systems, or shorter disease durations, can address the same central hypothesis. We have already seen that there has been poor translation of animal studies into the treatment of relapsing MS.^67^ It is important that we do better in finding treatments for progressive MS. The definition of EAE in NOD mice as a progressive model appears to be based on the trajectory of the mean disease scores, but it is clear from this study that this description is a misrepresentation of the disease course of individual mice. We have made the case previously that it is essential that more information such as maximum and minimum score and number of animals with disease be presented such that graphs of group disease scores can be better interpreted.^3^, ^68^ This, however, ultimately requires access to the raw data.

Access to the source data is being requested of clinical trials (www.clinicalstudydatarequest.com) to limit data hacking and hiding.^4^, ^69^ Although some journals are requesting statements that data are accessible, in the electronic age there is now no barrier to depositing raw data in workable spreadsheets. This is because a statement of supply of raw data can be hollow and unenforced. With deposition of raw data during submission of manuscripts such data can be interrogated by the reviewers and the readers. This would thus make the preclinical space more responsive and reproducible, as poor quality data are less likely to be submitted or published and can more quickly be challenged, avoiding the need to replicate studies.^3^, ^15^, ^68^ This is important as there is sometimes a lack of quality control in EAE studies where the data from control groups may be highly inconsistent and appears to sometimes fluctuate depending on whether an experimental‐treatment aims to find an augmentation or inhibition of disease.^70^, ^71^ This, coupled with poor‐reporting, notably of bias reduction, and data handling that can influence outcomes can lead to overinterpretation of data that is probably of marginal biological significance.^3^, ^62^, ^67^, ^72^, ^73^ Experimental data lacking quality control are not likely to be reproducible between laboratories, let alone between other strains/species and importantly have translational value for human studies.^3^ There have been many translational failures and only two of the 15 licensed treatments for MS had their origins in preclinical MS studies, although the majority of the other licensed treatments have subsequently been found to reduced EAE severity.^15^, ^67^, ^73^ It is therefore important that preclinical studies are used in a way to reflect the clinical indication, if they are to have translational value.^3^, ^15^, ^73^ Given the drive to perform more humane animal studies, notably to limit severe procedures in animals, there is no place for science that causes unnecessary animal suffering. Learned societies and governments need to lead journals to universally adopt transparent data deposition if animal‐based translational neuroscience is to remain a credible part of research.

## Conflict of Interest

There is nothing relevant to declare.

## Author Contribution

Concept: DB, SA; Funding: SA, AC, DMO; Data Acquisition: SA, KOS, DMO. Data Analysis & presentation: DB, SA, EM; Manuscript: All.

## Supporting information


**Data S1.** Individual daily disease scores of animals
